# Multilocus sequence types of Finnish bovine *Campylobacter jejuni *isolates and their attribution to human infections

**DOI:** 10.1186/1471-2180-10-200

**Published:** 2010-07-26

**Authors:** Caroline PA de Haan, Rauni I Kivistö, Marjaana Hakkinen, Jukka Corander, Marja-Liisa Hänninen

**Affiliations:** 1 Department of Food Hygiene and Environmental Health, University of Helsinki, Agnes Sjöberginkatu, Helsinki, Finland; 2 Research Department, Finnish Food Safety Authority, Mustialankatu, Helsinki, Finland; 3 Department of Mathematics and Statistics, University of Helsinki, Gustaf Hällströmin katu, Helsinki, Finland; 4 Department of Mathematics, Åbo Akademi University, Aningaisgatan, Åbo, Finland

## Abstract

**Background:**

*Campylobacter jejuni *is the most common bacterial cause of human gastroenteritis worldwide. Due to the sporadic nature of infection, sources often remain unknown. Multilocus sequence typing (MLST) has been successfully applied to population genetics of *Campylobacter jejuni *and mathematical modelling can be applied to the sequence data. Here, we analysed the population structure of a total of 250 Finnish *C. jejuni *isolates from bovines, poultry meat and humans collected in 2003 using a combination of Bayesian clustering (BAPS software) and phylogenetic analysis.

**Results:**

In the first phase we analysed sequence types (STs) of 102 Finnish bovine *C. jejuni *isolates by MLST and found a high diversity totalling 50 STs of which nearly half were novel. In the second phase we included MLST data from domestic human isolates as well as poultry *C. jejuni *isolates from the same time period. Between the human and bovine isolates we found an overlap of 72.2%, while 69% of the human isolates were overlapping with the chicken isolates. In the BAPS analysis 44.3% of the human isolates were found in bovine-associated BAPS clusters and 45.4% of the human isolates were found in the poultry-associated BAPS cluster. BAPS reflected the phylogeny of our data very well.

**Conclusions:**

These findings suggest that bovines and poultry were equally important as reservoirs for human *C. jejuni *infections in Finland in 2003. Our results differ from those obtained in other countries where poultry has been identified as the most important source for human infections. The low prevalence of *C. jejuni *in poultry flocks in Finland could explain the lower attribution of human infection to poultry. Of the human isolates 10.3% were found in clusters not associated with any host which warrants further investigation, with particular focus on waterborne transmission routes and companion animals.

## Background

*Campylobacter jejuni *is the most common bacterial cause of human gastroenteritis worldwide [1]. In many European countries, including Finland, the number of laboratory confirmed *C. jejuni *infections doubled in the last decade [2]. In Finland, approximately 4500 cases were reported in 2008 [3], with an incidence of 85/100 000 inhabitants.

*Campylobacter *outbreaks are relatively uncommon in industrialized countries, and most of the cases occur sporadically [1]. As a consequence, the sources of infection remain mostly unknown. Epidemiological studies in different countries indicate that eating improperly cooked meat and handling chicken carcasses are important risk factors for acquiring the illness [1,4]. Other risk factors highlighted in epidemiological studies include contact with pets [5], drinking untreated water [4] and swimming in natural water sources [6]. Outbreaks of campylobacteriosis are most commonly associated with drinking unpasteurized milk or contaminated water [7,8] and eating improperly cooked poultry meat [9].

*C. jejuni *has a wide distribution among different warm-blooded animals, including poultry, bovines, pigs, cats, dogs and various wild animals [10,11] and birds. As a consequence of faecal contamination, *C. jejuni *is also frequently isolated from natural waters [12]. To estimate the proportion of human infections attributed to different sources of infection, various typing methods have been applied to distinguish between strains. Pulsed field gel electrophoresis (PFGE) has been considered the method of choice due to its high discriminatory power; however, during the last decade - after its description for *C. jejuni *- multilocus sequence typing (MLST) [13] has generally been accepted as the most suitable method for population genetic analyses. The major advantages of MLST compared to PFGE are the standardized nomenclature and the ability to easily transfer and compare results between laboratories worldwide. Furthermore, different mathematical modelling approaches can readily be applied on the resulting sequence and allele data to facilitate source attribution. For this purpose, different Bayesian approaches, inferring the genetic population structure of *C. jejuni*, have garnered the most interest [14-17]. Bayesian Analysis of Population Structure (BAPS) [18-21] has recently been successfully applied in inferring population structures of *E. coli *[22] and the *S. mitis *group streptococci [23]. BAPS showed, in a simulation study, comparable power to other methods and was deemed also to be highly efficient from computational perspective [24].

Limited data exists on sequence types (STs) present among bovine isolates in Finland [25], and estimating the proportion of human infections potentially linked to this source has been difficult. To better understand the diversity of Finnish bovine *C. jejuni*, we characterized 102 isolates using MLST. We used BAPS v. 5.3 for source attribution purposes and included additional MLST data obtained in our previous study [25] from Finnish bovines, retail poultry meat and human isolates from 2003.

## Results

### MLST of bovine isolates

Genotypes of a total of 102 bovine *C. jejuni *isolates were identified by nucleotide sequences at all seven MLST loci. Ninety-three of these were assigned into nine previously described clonal complexes (CCs) (Table 1). The ST-21 CC was predominant (51%), followed by the ST-61 CC (17.6%), the ST-45 CC (10.8%), the ST-48 CC (4.9%) and the ST-677 CC (2.9%). Of the 50 STs observed among the isolates, 23 (46%) were novel. Thirty-two isolates (31.4%) had a unique ST, and the most common STs among the isolates were ST-53 (12.7%), followed by ST-61 (7.8%) and ST-883 (6.9%).

**Table 1:** Distribution of multilocus sequence types among our bovine *Campylobacter jejuni *isolates from 2003.

		**Allele no**.						
		
CC	ST	*aspA*	*glnA*	*gltA*	*glyA*	*pgm*	*tkt*	*uncA*
ST-21 CC	21 (3)	2	1	1	3	2	1	5
	43	2	1	5	3	4	1	5
	50 (4)	2	1	12	3	2	1	5
	53 (13)	2	1	21	3	2	1	5
	141	2	1	10	3	2	1	5
	262 (2)	2	1	1	3	2	1	3
	333 (2)	2	1	21	2	2	1	5
	451 (4)	2	1	2	3	2	3	5
	561	2	1	21	4	2	1	5
	761	2	1	1	4	2	1	5
	883 (7)	2	17	2	3	2	1	5
	1459	2	1	1	2	2	1	5
	1823	2	1	177	3	2	1	5
	1952	2	1	12	3	1	1	5
	**2956**	2	17	2	2	2	1	5
	**2957 (2)**	2	1	1	3	**393**	**318**	5
	**2958**	2	1	12	3	2	20	5
	**2959**	2	1	2	137	2	3	5
	**2996 (2)**	2	1	2	4	2	3	5
	**3352**	2	1	2	2	2	3	5
	**3788**	4	1	6	3	2	1	5
	**3810**	14	4	1	3	19	1	5
								
ST-22 CC	**3892**	1	3	6	3	3	3	3
								
ST-42 CC	42	1	2	3	4	5	9	3
								
ST-45 CC	45 (3)	4	7	10	4	1	7	1
	97	4	7	10	4	1	1	1
	230	4	7	41	4	42	7	1
	242 (2)	4	7	10	2	1	7	1
	1701	4	7	10	4	1	51	1
	**2663 (2)**	4	7	10	3	1	7	1
	**3357**	4	7	10	3	42	51	1
								
ST-48 CC	475 (3)	2	4	1	4	19	62	5
	**2955**	2	4	1	2	19	62	5
	**3893**	2	4	2	2	7	51	5
								
ST-61 CC	61 (8)	1	4	2	2	6	3	17
	618 (3)	1	4	2	2	6	3	5
	820	1	4	2	4	6	3	17
	**2974**	1	4	2	3	2	3	**234**
	**3351 (3)**	1	4	2	3	6	3	17
	**3509**	1	4	2	4	6	3	38
	**3894**	10	4	2	3	6	3	17
								
ST-206 CC	**3360**	2	17	5	4	2	1	5
								
ST-658 CC	**3000**	2	4	2	4	19	1	8
								
ST-677 CC	677 (3)	10	81	50	99	120	76	52
								
Unassigned	58	19	24	23	20	26	16	15
	586 (4)	1	2	42	4	98	58	34
	**2961**	1	17	2	4	2	3	5
	**2999**	2	2	107	4	120	76	1
	**3354**	2	2	42	4	98	58	5
	**3787**	1	4	1	4	19	62	5

Numbers in parentheses after each ST denote the number of isolates. New STs and alleles are shown in boldface.

### Analyses of population structure of Finnish bovine, poultry and human isolates

In our total set of 250 bovine, poultry and human isolates, including data from our previous study [25], 74 STs were found and included in the population structure analysis. The 74 STs were found among 13 CCs and the most common CCs were the ST-21 CC (39.6%), the ST-45 CC (30.0%), the ST-61 CC (8.0%), the ST-677 CC (4.4%) and the ST-48 CC (2.4%). The predominant STs were ST-45 (23.2%), ST-50 (16.8%), ST-53 (6.4%), ST-61 (4%) and ST-883 (3.6%).

Overall, 13.3% and 87.8% of the STs found in bovine and poultry isolates, respectively, were also found in human isolates. Conversely, 72.2% and 69% of the STs found in human isolates were also found in bovine and poultry isolates, respectively. Furthermore, 81.8% of the STs found in poultry were found in bovine isolates, but only 7.5% of those in bovines were present in poultry isolates.

In analysing the relationships of clonal complexes with hosts, the ST-21 (p < 0.01) and ST-61 CCs (p < 0.0001) were associated with bovine isolates, whereas the ST-45 CC was associated with poultry (p < 0.0001) and human isolates (p < 0.001). Bovine isolates were found in bovine-associated CCs in 65.8% of the cases. Poultry and human isolates were found in the ST-21 CC in 15.1% and 36% of the cases, respectively. The ST-61 CC did not occur among poultry and human isolates. The ST-45 CC contained 69.7% of all the poultry isolates, 40.2% of the human isolates and 10.8% of the bovine isolates.

ST-61 (p < 0.001), ST-53 (p < 0.0001), ST-58 (p = 0.01), ST-451 (p = 0.02) and ST-883 (p = 0.001) were associated with the bovine host and contained 38.3% of the bovine isolates. None of the human or poultry isolates represented bovine-associated STs. ST-45 was associated with poultry (p < 0.0001) and human isolates (p < 0.01) and was found in 66.7% of the poultry isolates, 32% of the human isolates and 4.2% of the bovine isolates. ST-50 was associated with human isolates (p < 0.0001) and was found in 34% of the human isolates, 15.1% of the poultry isolates and 3.3% of the bovine isolates. ST-137 was associated with the human isolates (p < 0.01), but was absent from both other sources.

Using BAPS, nearly all estimation runs converged to the same solution with five clusters having high posterior certainty in its vicinity according to the program output. BAPS clusters 1 and 4 contained the majority of isolates (86.8%). BAPS cluster 1 contained all STs found in the ST-22, ST-45, ST-48, ST-283, and ST-658 CCs in addition to two significantly admixed STs in the ST-21 CC (Table 2). One ST of the ST-48 (ST-2955) and ST-658 CCs (ST-1967) was admixed as well. BAPS cluster 2 contained a total of three unassigned STs which were only found in human isolates. In BAPS cluster 3 the ST-677 CC was grouped together with two uncommon, unassigned STs. BAPS cluster 4 comprised all, but two, STs of the ST-21 CC, all STs from the ST-52, ST-206, ST-257 and ST-1287 CCs and one ST (ST-618) from the ST-61 CC, which was significantly admixed. The remainder of the ST-61 CC formed a distinct cluster (cluster 5), with no admixed STs and contained only bovine isolates.

**Table 2:** Distribution of clonal complexes and sequence types accordingly BAPS clusters.

			Host		
			
BAPS cluster	CC	ST	Bovine	Chicken	Human
1	21	**1952**	1	0	0
		**3810**	1	0	0
	22	1966	0	0	1
		3892	1	0	0
	42	42	1	0	0
	45	45	5	22	31
		97	1	0	0
		137	0	0	6
		230	1	0	0
		242	2	0	0
		1701	1	0	0
		1964	0	0	1
		1971	0	1	0
		1973	0	0	1
		2663	2	0	0
		3357	1	0	0
	48	475	4	0	0
		**2955**	1	0	0
		3893	1	0	0
	283	267	0	1	0
	658	658	0	0	1
		**1967**	0	0	2
		3000	1	0	0
	UA	586	4	0	0
		**1962**	0	0	1
		3354	1	0	0
		3787	1	0	0
**Total**			**30**	**24**	**44**
					
2	UA	1959	0	0	1
		1960	0	0	1
		1961	0	0	1
**Total**			**0**	**0**	**3**
					
3	677	677	3	0	5
		794	0	1	2
	UA	1080	0	1	0
		2999	1	0	0
**Total**			**4**	**2**	**7**
					
4	21	21	4	0	1
		43	1	0	0
		50	4	5	33
		53	16	0	0
		141	1	0	0
		262	2	0	0
		333	2	0	0
		451	5	0	0
		561	1	0	0
		761	1	0	0
		883	9	0	0
		1459	1	0	0
		1969	0	0	1
		1823	1	0	0
		2956	1	0	0
		2957	2	0	0
		2958	1	0	0
		2959	1	0	0
		2996	2	0	0
		3352	1	0	0
		3788	1	0	0
	52	52	0	1	1
		305	0	0	1
	61	**618**	3	0	0
	206	46	0	0	1
		3360	1	0	0
	257	824	0	0	1
	1287	**945**	0	0	2
	UA	**58**	6	0	0
		1963	0	0	1
		**1970**	0	1	0
		1972	1	0	0
		1974	0	0	1
		2961	1	0	0
**Total**			**69**	**7**	**43**
					
5	61	61	10	0	0
		820	1	0	0
		2974	1	0	0
		3351	3	0	0
		3509	1	0	0
		3894	1	0	0
**Total**			**17**	**0**	**0**

STs shown in bold face were significantly admixed with at least one other cluster, but were put into the cluster with the highest posterior probability. Bold-faced underlined text shows number of isolates of each host in the specific BAPS cluster.

Admixture was mainly found in clusters 1 and 4 for a total of nine STs (12.2%) including a total of 18 isolates (7.2%). Mainly novel STs in the ST-21 complex (two STs), ST-48 complex (one ST), ST-658 complex (one ST), ST-1962 and ST-1970 were found to be admixed. However, also ST-618 (ST-61 CC), ST-945 (ST-1287 CC) and ST-58 (unassigned) were significantly admixed. Bovine isolates were found to be associated with admixture (p = 0.05).

BAPS clusters 4 and 5 were associated with the bovine isolates (Table 2), BAPS cluster 1 was associated with the poultry isolates and BAPS clusters 2 and 3 were not associated with any host. Bovine isolates were found in bovine-associated clusters in 71.7% of cases. Of the poultry isolates 72.7% were found in the poultry-associated cluster. Human isolates were found in the bovine-associated BAPS cluster 4 in 44.3% of the cases and in 45.4% of the cases found in the poultry-associated BAPS cluster 1.

**Figure 1:**
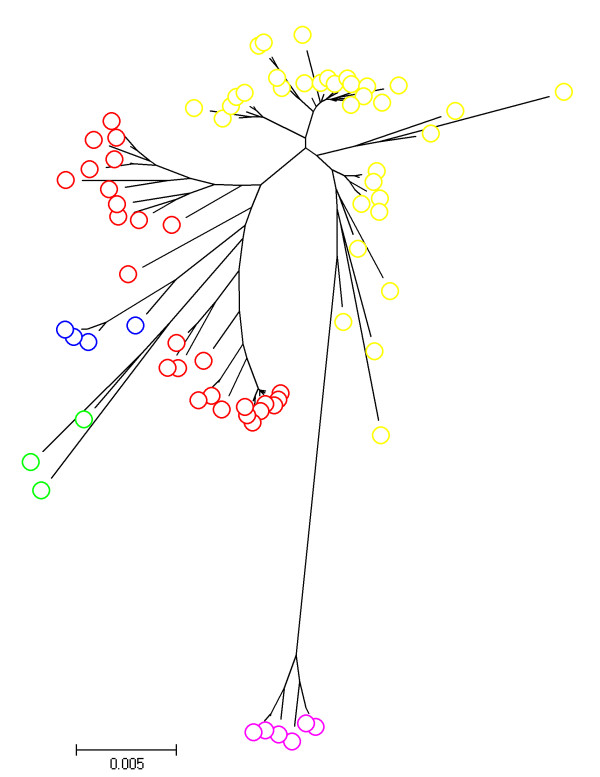
**Neighbour-joining tree illustrating BAPS clusters from a phylogenetic perspective**. BAPS cluster 1: Red; BAPS cluster 2: Green; BAPS cluster 3: Blue; BAPS cluster 4: Yellow; BAPS cluster 5: Purple.

The NJ tree shown in Figure 1 illustrates the molecular variation within and between the clusters estimated by BAPS from a phylogenetic perspective. eBURST analysis yielded seven groups containing two (smallest group) to 12 (biggest group) STs and 34 singletons. Table 3 shows the degree of similarity between the eBURST groups and BAPS populations. The biggest BAPS clusters (1 and 4) were made up of several eBURST groups, while BAPS cluster 2 did not have an equivalent eBURST group.

**Table 3:** Number of STs of *Campylobacter jejuni *assigned to both a BAPS population and an eBURST group

BAPS populations	eBURST groups						
	
	1	2	3	4	5	6	7
1	1	10			3		
2							
3							2
4	11		1	4		3	
5			5				

## Discussion

Our study revealed a high diversity of MLSTs among 102 bovine *C. jejuni *isolates obtained from three major Finnish slaughterhouses, representing 81 farms, in 2003. A total of 50 STs (nine CCs) were observed, nearly half of which were novel, emerging mostly from new combinations of known alleles and in two cases from new alleles carrying a one-nucleotide difference from alleles commonly found in cattle (*pgm *allele 2, *tkt *allele 1 and *uncA *allele 17). The emergence of a high number of novel STs could be explained by the life cycle of dairy cattle, providing a *C. jejuni *strain with the opportunity for long-lasting colonization and adaptation in the bovine host. However, re-infection with a different strain or multiple strains, and thus the occurrence of recombination events, cannot be excluded. The distribution of *C. jejuni *genotypes has previously been shown not to be random among farms, with farms no more than 1 km apart appearing to possess similar *C. jejuni *genotypes [12,26], supporting the persistence of clones in cattle herds. Probably due to the disperse distribution of farms in Finland, we found no clear evidence of regional differences in the distribution of bovine STs or CCs between different parts of the country. This is in agreement with findings from Scotland [27].

In this study, as well as previous studies, the ST-21 and ST-61 CCs were shown to be common in cattle [10,28]. The ST-61 CC, in particular, is strongly associated with bovines and has been observed in cattle in other studies worldwide [10,12,15,28-33]. We did not find members of the ST-61 CC in poultry or humans [25], and other studies have infrequently observed this CC in these hosts [28,31,32,34]. Also, ST-58 was one of the most prevalent bovine STs (5%) in our study, and STs that share five or more alleles with ST-58 (e.g. ST-2683, ST-3098, ST-3365, ST-3426, ST-3432 and ST-3443), have previously been reported only from cattle in the UK and Ireland [35] and Scotland [27]. In addition to STs in the ST-61 CC, ST-58 may represent another clonal lineage of *C. jejuni *adapted to the bovine gut.

Source attribution is an important task in the risk assessment of the impact of different potential reservoirs for human infections caused by *C. jejuni*, and MLST has been shown to be an efficient method for assessing clusters of isolates with host specificity [36]. On clonal complex level 65.8% of the bovine isolates were found in bovine-associated CCs and 69.7% of the poultry isolates were found in poultry-associated CCs. However, on ST level only 38.3% of the bovine isolates were found in bovine-associated STs, reflecting the high diversity of STs found in bovine isolates within clonal complexes. In addition, we used BAPS, a tool that has recently become popular for inferring population genetic structure [18,19,21] to assign our isolates to genetically differentiated groups. BAPS divided the 74 STs into five clusters such that clusters 1 and 4 contained all STs which BAPS identified as mosaics due to recombination. Of the bovine isolates 71.7% were found in the bovine-associated BAPS clusters 4 and 5. Similarly, poultry isolates were found in 72.7% of the cases in the poultry-associated BAPS cluster 1. These results indicate that BAPS was useful for host assignment, even though our dataset was relatively small. BAPS analysis showed comparable power to host assignment using clonal complexes but also reflected the phylogeny of our data.

BAPS clusters 1 and 4 contained most of the isolates and were substantially heterogeneous in the distribution of clonal complexes. Especially, the poultry-associated BAPS cluster 1 was very heterogeneous; the ST-45 CC was most common and grouped together with several uncommon, unrelated clonal complexes, often not found in our poultry isolates. In our previous study [25], the ST-45 CC found in our human isolates was associated with tasting of raw or undercooked meat as well as contact with dogs or cats. Also, the ST-45 CC has been found from penguins on the Antarctic [37], implying that this CC has a wide host range and is environmentally well adapted. The ST-22, ST-42 and ST-48 CCs, which were grouped together with the ST-45 CC in BAPS cluster 1, have been commonly found in companion animals in other studies [11,28,38]. However, more studies are needed to establish the role of environmental contamination sources serving as *C. jejuni *vectors for both human infection and chicken colonization.

Most admixture was found in clusters 1 and 4 with the majority of admixed STs being novel and associated with the bovine isolates. All admixed STs with the highest posterior probability in cluster 1 (poultry-associated) were admixed with cluster 4 (bovine-associated) and most of these STs were found only in bovine isolates. In contrast, most admixed STs with the highest posterior probability in cluster 4 were admixed with clusters 2 and 3, in which only human isolates were assigned to and mostly contained uncommon, unassigned STs. These findings could imply that recombination is more common in STs specific to bovines, which is supported by the high diversity of our bovine isolates. Bovines have a longer life-span than poultry and persistence of *C. jejuni *clones in herds and specific bovine-associated lineages imply that these strains can adapt to long-lasting colonization, thereby increasing the chance of horizontal transfer of genetic material and recombination.

The ST-61 CC was found as a separate cluster (cluster 5) by BAPS, with the exception of ST-618 (cluster 4, admixed with cluster 1). This finding was not surprising since the ST-61 CC is known to have imported *C. coli *alleles (e.g. *uncA17*) and therefore is phylogenetically less related to other *C. jejuni *clonal complexes [39]. Both ST-618 and ST-3509 do not possess the *uncA17 *allele, but ST-3509 carries the *uncA38 *allele. This allele is common in both the ST-61 CC and the *C. coli *related ST-828 CC and likely the presence of this allele caused ST-3509 to be included in BAPS cluster 5. ST-618, however, carries the *uncA5 *allele, which is commonly found in both the ST-21 CC (cluster 4) and the ST-48 CC. This explains why this particular ST was grouped together with the ST-21 CC and at the same time admixed with cluster 1. These results demonstrate that the import of *C. coli *DNA can have a large impact on the MLST analysis of *C. jejuni *strains and this should be taken into account in source attribution studies.

Studies from the UK [15-17] and New Zealand [14]; have indicated that chicken (poultry) could attribute to 57-80%, cattle (and sheep) to 18-39%, and other, mainly environmental, sources to 1-4% of all *C. jejuni *infections. Compared to these studies we found a lower source attribution for chickens (45.4%) and a higher source attribution for bovines (44.3%). This could be the result of limited sampling of *C. jejuni *isolates from chicken meat in our study and the fact that *C. jejuni *is more difficult to detect by cultivation from meat compared to faecal samples. The meat samples, however, represented all three major chicken meat producers and were collected during the summer peak [25], when most human *C. jejuni *infections occur in Finland [3]. The national low prevalence of *Campylobacter *spp. in Finnish chicken flocks (6.5% in 2003) [2] in comparison to other EU countries could lead to a different source attribution when compared to studies from other countries. In a Finnish slaughterhouse study, *C. jejuni *was detected in 19.5% of the faecal samples and 3.5% of bovine carcasses [40]. However, none of the *C. jejuni *isolates from carcasses represented PFGE types similar to human isolates [41]. Bovines could be an underestimated route for *Campylobacter *infections in Finland, although foodborne transmission would be least likely. However, transmission could occur through either direct contact or environmental transmission by shared reservoirs for human patients and bovine *C. jejuni *strains.

A large proportion of our isolates (10.3%) could not be attributed to any source (BAPS clusters 2 and 3). More than half of these isolates represented the ST-677 CC, which has been detected in various hosts, including starlings [42], rabbits, environmental waters, wild birds and cattle [10]. In our previous study this CC was related to drinking non-chlorinated water from a small water plant or from natural water sources [25]. Faecal contamination from wild animals and birds into natural water sources is common and could be hypothesized to have a pronounced role in human infections in summer in our Finnish study region Uusimaa. This is also supported by the Finnish case-control study that identified swimming and drinking from dug wells as important risk factors for infection during summertime [6]. Therefore the role of different water-associated transmission routes should not be underestimated in future attribution studies of Finnish domestically acquired *C. jejuni *human infections.

## Conclusions

Due to the wide distribution and occurrence of some *C. jejuni *CCs and STs among different hosts, source attribution is a complicated issue and Bayesian methods are considered useful for quantitative probabilistic assignment of STs to genetically related clusters. In our study 71.7% of the bovine isolates and 72.7% of the poultry isolates were found in clusters associated with each host. Of the human isolates 44.3% was found in the bovine-associated BAPS cluster 4 and 45.4% was found in the poultry-associated cluster. Inclusion of MLST data in detailed epidemiological case-control studies and parallel extensive regional sampling schemes would greatly improve the attribution of human infections to the source and help develop specific control schemes to limit the numbers of human infections.

## Methods

### Bovine isolates

A total of 102 *C. jejuni *isolates from bovine rectal samples isolated in a survey on *Campylobacter *spp. in Finnish cattle at slaughter in 2003 [40] were included in this study. The isolation method included an enrichment stage in Bolton broth and subcultivation on mCCDA as described by Hakkinen *et al*. [40]. Sampling was performed over a 12-month period, and the frequency of sampling was determined on the basis of the numbers of cattle slaughtered in each slaughterhouse to ensure that the collection of isolates would represent the bovine *C. jejuni *population in these slaughterhouses. The isolates originated from clinically healthy cattle from 81 farms in 5 of the 6 Finnish counties. They were isolated in three slaughterhouses: one located in the western and two in the eastern part of Finland. Isolates were stored deep-frozen at -70°C in skimmed milk or Brucella broth with 15% glycerol.

### DNA extraction

The isolates were cultured on Brucella agar (BBL, Becton Dickinson, MD, USA) with 5% bovine, horse or sheep blood and incubated under microaerobic conditions at 37°C for 48 h. The DNA was isolated with the Wizard^® ^Genomic DNA Purification Kit (Promega, WI, USA), diluted to 10 ng/μl and stored at -20°C.

### Multilocus sequence typing (MLST)

MLST was performed according to the method described by Dingle et al [13]. The primers and settings are described on the PubMLST website [35]. In addition, alternative primers described previously [38,43] were used. In the event of unsuccessful PCR with the primer sets in these schemes, other primer combinations were chosen, and the annealing temperatures were adjusted if necessary. MultiScreen PCR plates (Millipore, MA, USA) were used to purify the PCR products. Sequencing reactions were carried out by using the BigDye terminator v. 3.1 Ready Reaction Cycle Sequencing Kit (Applied Biosystems Inc., CA, USA). The Agencourt ^®^CleanSEQ kit (Beckman Coulter Genomics, Takeley, United Kingdom) was used for cleaning the reactions. The sequencing products were run on an ABI3130XL Genetic Analyzer or an ABI3730 DNA analyzer (Applied Biosystems, Foster City, CA, USA). The sequences were assembled using the Staden package [44] or the assembler implemented in BioNumerics v. 5.1 software. Allele numbers, STs and CCs were assigned using the PubMLST database [35]. New alleles and STs were submitted to the database.

### Analysis of population structure and host assignment

The Bayesian program BAPS v. 5.3 [18,19,21], was used to investigate the population genetic structure by clustering STs into genetically differentiated groups and evaluating them to predict the sources of human campylobacteriosis. For the analysis, the sequences of the STs of bovine isolates in this study was combined with those of ST types of 18 bovine, 33 poultry meat as well as 97 patient isolates from domestically acquired infections collected at the Helsinki University Central Hospital Laboratory from the Helsinki-Uusimaa area in 2003 described in our previous MLST study [25]. Linkage clustering and the corresponding admixture model were used [18-21]. The estimation algorithm was used with 10 replicate runs where the maximum number of clusters was set to values in the interval 2-10 and STs were assigned to clusters with the highest posterior probability. Admixture inference was based on 100 Monte Carlo runs and 100 Monte Carlo reference samples to estimate the p-values. Significant admixture was set at a threshold level of *P *≤ 0.05 to detect admixed STs. To gain further insight into the BAPS derived clusters, we did a phylogenetic analysis of the STs using software MEGA v 4.0.2 [45]. A neighbour-joining (NJ) tree based on maximum composite likelihood for concatenated allele sequence data was generated and the BAPS clusters were mapped on the tree. eBURST analysis [46] of the 74 STs in our dataset was performed using default options in eBURST version 3 available at http://eburst.mlst.net[47].

### Statistical analyses

Analyses of association of each BAPS cluster, and ST or CC with the source of isolation were carried out using the Chi-square or Fisher's exact two-tailed test when appropriate. Results were considered statistically significant at *P *≤ 0.05.

## Authors' contributions

CPAdH performed MLST analyses and drafted the manuscript. RIK constructed the study design and aided in drafting the manuscript. MH identified the bovine isolates and aided in the study design. JC performed all mathematical analyses and assisted in drafting the manuscript. MLH conceived the study idea, participated in the design and helped drafting the manuscript. All authors read, commented and approved the manuscript.
